# Excitatory Dorsal Lateral Prefrontal Cortex Transcranial Magnetic Stimulation Increases Social Anxiety

**DOI:** 10.3390/brainsci13070989

**Published:** 2023-06-24

**Authors:** Anthony Minervini, Adriana LaVarco, Samantha Zorns, Ruth Propper, Christos Suriano, Julian Paul Keenan

**Affiliations:** Cognitive Neuroimaging Laboratory, Montclair State University, Montclair, NJ 07043, USA; minervinia2@montclair.edu (A.M.); surianoc@montclair.edu (C.S.)

**Keywords:** social exclusion, DLPFC, TMS, rTMS, pain, social pain, Cyberball

## Abstract

Social exclusion refers to the experience of rejection by one or more people during a social event and can induce pain-related sensations. Cyberball, a computer program, is one of the most common tools for analyzing social exclusion. Regions of the brain that underlie social pain include networks linked to the dorsal lateral prefrontal cortex (DLPFC). Specifically, self-directed negative socially induced exclusion is associated with changes in DLPFC activity. Direct manipulation of this area may provide a better understanding of how the DLPFC can influence the perception of social exclusion and determine a causal role of the DLPFC. Transcranial magnetic stimulation (TMS) was applied to both the left and right DLPFC to gauge different reactions to the Cyberball experience. It was found that there were elevated exclusion indices following right DLPFC rTMS; participants consistently felt more excluded when the right DLPFC was excited. This may relate to greater feelings of social pain when the right DLPFC is manipulated. These data demonstrate that direct manipulation of the DLPFC results in changes in responses to social exclusion.

## 1. Introduction

Social organisms, including humans, have evolved to form complex social bonds to secure necessities such as shelter and resources [1,2]. Social interactions that involve exclusion of an individual from a group can trigger a threat response, as well as have a direct impact on the mental health of the individual [3,4,5,6]. Individuals may label social scenarios as positive or negative experiences, which can lead to feelings of loneliness if the individual was excluded [7]. Exclusion experiences can occur during a physical social exclusion event or a virtual representation of one via computer programs [8].

Cyberball, a computer program that replicates firsthand exclusion events, can be used to evaluate human interactions with ostracism [9,10,11,12]. The program utilizes a virtual game of catch to implement firsthand exclusion. Employing this, research has investigated a critical social phenomena.

For example, when social connections are threatened, mortality rates increase [13,14,15,16]. A 2006 study reported that socially isolated children were at increased risk for health problems such as cardiovascular disease [15]. Other risk factors, such as socioeconomic status and general health, did not contribute to the increased rates of mortality and cardiovascular disease. These findings underscored the value of social connections by showing how isolation during critical human developmental stages can have a negative influence on health over a lifespan [15,17].

### 1.1. The Right Dorsal Lateral Prefrontal Cortex

Social exclusion may elicit DLPFC responses within the pain-related network [18,19,20,21]. The DLPFC is responsible for many different roles, one of which may involve the regulation of pain in both a physical and emotional regard. This idea is supported by research showing that there is greater activation in the DLPFC when remembering events such as abandonment [19]. Studies suggest that the DLPFC displays similar responses to actual pain and potential pain by activating what has been termed the pain matrix [22,23,24]. The pain network consists of the insula, cingulate cortex, and somatosensory cortices [22,25]. In both physical and social rejection, there is an increase in activity in the dorsal anterior cingulate cortex (dACC) and the anterior insula, which are regulated by the DLPFC in the pain control network [20,26,27,28]. This highlights the importance of the DLPFC and how regulation of these processes is conducted by the DLPFC, thus signifying the area as a key region for the feeling of pain when social exclusion occurs.

In addition to the pain matrix, the DLPFC is connected to the top-down control of other behaviors, such as the regulation of emotions [29,30,31,32]. Furthermore, the regulation of emotions in the DLPFC can influence the amount of distress experienced in social settings [31]. Interestingly enough, not only does the DLPFC regulate the amount of distress experienced, but it is believed that sensitivity to rejection is processed within this brain region [33]. This idea is further supported by studies that measure rejection and rejection detection in terms of social scenarios [34]. It has been suggested that social rejection activates detection processing in the brain [35,36]. While previous research has focused on the PFC in regard to isolation, we intended to target the DLPFC to further evaluate social context and utilize Cyberball as a firsthand exclusion invoker.

The DLPFC has been shown to play a crucial role in the control of emotions [37,38,39]. Utilizing either stimulating or inhibiting TMS procedures on the left and right DLPFC may result in differing effects in terms of social decision making and exclusion scenarios, a finding based on a meta-analysis of TMS studies and the DLPFC [40,41,42]. For example, fairness and selfishness in social interactions have been found to be influenced by disruptive low frequency TMS, such as 1 Hz of the DLPFC, and more specifically the right DLPFC [43,44,45]. It was found that inhibition of the right DLPFC correlated with increased selfishness [45]. Further research revealed that right DLPFC disruption did not significantly affect assessments of fairness and that fairness perception may be independently represented [44,45]. Additionally, increased right prefrontal cortical activity may be associated with withdrawal, or primarily negative emotions, and left hemisphere prefrontal cortical activity with approach, or primarily positive emotions, suggesting a potential differential response of the hemispheres to social exclusion (See Spielberg et al., 2011, for review) [46].

### 1.2. TMS and rTMS

Transcranial magnetic stimulation (TMS) is a non-invasive technique for stimulating different brain regions. Low frequency TMS (1 Hz) inhibits brain activity, while a higher frequency such as 10 Hz is excitatory. TMS has been clinically used for various conditions, such as anxiety and depression [47]. Typically, TMS and rTMS are performed on the left side of the brain for depression treatments; more specifically, the left prefrontal areas are targeted daily [48]. Patients who undergo TMS treatment for depression usually attend sessions for around 4–6 weeks, although in some cases patients may require additional weeks depending on the condition [49,50]. Ultimately, patients experienced a dramatic decrease in depression levels around the four-week mark [48,51].

TMS is utilized to treat anxiety, but it can also considerably increase anxiety levels among individuals with underlying factors such as panic disorder [52]. TMS can generate changes within brain networks to help with anxiety, similar to its beneficial effect on depression when used routinely [53]. Studies support the idea that the use of low frequency rTMS in the right DLPFC can lessen the effects of anxiety [54,55,56]. The consensus is that inhibitory rTMS can show favorable outcomes in disorders such as generalized anxiety disorder and panic disorder, along with other conditions [57,58,59].

A 2020 study used TMS to explore social pain using pictures while targeting the ventrolateral prefrontal cortex (VLPFC) [60]. This study supports the notion that emotional social regulation is a function of the VLPFC [61,62]. Applying 10 Hz rTMS to the right DLPFC, as well as the right VLPFC, relieved social pain [63]. Furthermore, relief was measured through distraction, which relates to the concept that the DLPFC is involved in attention during early stages of emotional regulation [38]. Additional research demonstrates that the same region of the brain in non-human primates also correlates with social situations [64]. These and other studies support the idea that social interactions, and the various components thereof, strongly recruit the prefrontal cortex, which justified the use of TMS to further investigate these relationships.

As previously indicated, social exclusion can trigger the same brain pathways as physical pain [22]. Current literature supports that there may be overlapping brain circuitry between physical and emotional pain [22]. For example, some patients who experience somatoform pain disorders with no medical explanation had also reported higher levels of traumatic social events [65]. Similarly, exclusion events have been correlated with higher sensitivity to physical pain. For example, there were increased pain levels when participants immersed their hands in ice water following an exclusion event, compared to ice water immersion without exclusion [66,67]. Furthermore, a growing body of research has shown that social support helps reduce feelings of physical pain, suggesting that influences on either social or physical aspects of life have impacts on one another [22,68].

During childhood, social rejection can also trigger more aggressive behavior [69]. Social scenarios that are particularly negative can increase aggression, and in fact aggression may have a linear relationship with the severity of isolation or bad experiences [5,70]. Moreover, results support the idea that there are key activations within the DLPFC and VLPFC when assessing social rejection, further supporting the claim that social processing and pain are located within these regions [20,71].

In addition to ostracism, the fear of being ignored before an event is an equally powerful feeling [72]. A 2003 study suggests that the idea of being excluded from a social event is physically painful [73]. Furthermore, those who have experienced social rejection tend to report higher-than-average levels of depression [74]. Depression has also been reported in older adults as a direct cause of social isolation [75]. This ties in well with the fact that TMS is now commonly employed to clinically treat depression by specifically targeting the DLPFC [51,76,77,78].

The DLPFC is one region in a network involved in the experience of painful social exclusion. For example, the neighboring dorsal anterior cingulate cortex (dACC) is an additional brain region implicated in the neurological response to social exclusion [22]. The dACC, which is activated in response to both physical pain and social exclusion, is thought to aid in the treatment of emotional and social pain [20].

### 1.3. Cyberball: Creating Exclusion

Manipulation of social exclusion can be completed in various ways. For exclusion scenarios, some of the most reliable studies use a program called Cyberball. Cyberball is a computer program that allows users to be excluded from a virtual game through a pre-programmed social situation [8]. Participants are excluded from being thrown a ball after a set number of throws, which then results in the ostracizing scenario. The number of tosses, length of game, and number of participants are commonly manipulated variables. 

### 1.4. Gap in Literature and Hypothesis

The DLPFC, specifically the right DLPFC, plays a role in pain, emotion, and social decision making. While numerous Cyberball experiments have been conducted, there is a lack of research when it comes to directly manipulating brain function to detect a direct causal link to feelings of social exclusion. A 2021 study provided evidence that targeting the DLPFC as well as the VLPFC improved the ability to regulate emotions [63]. Additionally, cognitive control is developed in these regions of the brain and is suggested to have a role in attention given to social situations [79,80]. Therefore, while there have been studies that apply 10 Hz rTMS to the DLPFC and PFC, such as the 2021 study conducted by Zhao et al., it was not performed with a firsthand exclusion game such as Cyberball [63]. Thus, the goal of our study was to influence the responses to firsthand social exclusion and explore a direct causal role of the DLPFC. We hypothesized that 10 Hz rTMS applied to the right DLPFC would cause increased negative feelings of social exclusion.

## 2. Materials and Methods

### 2.1. Participants

Thirteen participants were recruited for the study through social media, word-of-mouth, and a brochure handed out on the Montclair State University campus. The participants’ ages ranged from 18 to 65, with 4 males, 8 females, and 1 identifying as ‘other’. Participant self-reported ethnicities included 6 Hispanic, 1 Asian, 4 Caucasian, and 2 who declined to answer. Two participants self-reported being left-handed and 11 right-handed. In exchange for their participation in the experiment, the participants were paid $25. This study was approved by the Institutional Review Board at Montclair State University (IRB #22-23 424), and all participants were treated ethically and per APA guidelines. Previous studies with similar sample size resulted in medium power at eta^2^ = 0.40 [81,82,83,84].

### 2.2. Materials

All TMSs employed a 7 cm figure-of-eight coil and a Magstim 200 rapid stimulator to deliver pulses at both 10 Hz and 1 Hz. All presentations, excluding the virtual game of catch, were performed on a Lenovo Thinkpad T490 using Testable. Cyberball 5 empirisoft software was downloaded and installed to implement the exclusion game (www.empirisoft.com/cyberball.aspx, accessed on 5 June 2022). Using both Trigno wireless MEP amplifiers and DelSys software, the motor threshold (MT) of each participant was determined (Delsys; www.delsys.com, accessed on 5 June 2022). For the duration of the experiment, the participants wore a Lycra swim cap as well as earplugs.

### 2.3. Stimuli

Following the conclusion of the Cyberball game, participants were asked a series of questions specific to their experience while playing the game. A questionnaire was constructed using portions of a question bank made specifically for Cyberball. Williams provided questions based on senses of belonging, self-worth, control, and meaningful existence [85], while certainty questions were created by Hales and Williams [86]. Additional questions were written by the research team and placed into Testable. The categories of questions consisted of control, mood, certainty, belonging, self-esteem, anxiety, and the perception of others. These sets were divided into reflexive and reflective scales that referred to how participants felt during (reflexive) or after (reflective) the game. Questions were pulled from a pool, and answers were given using a slider response bar that ranged from 1 to 100. All other numbers were not visible, and the slider started at the neutral position of 50. Questions used are in Table 1 and Table 2.

### 2.4. Procedure

Prior to the experiment, each participant provided informed consent. Wassermann’s guidelines were followed to determine threshold [86]. Before TMS was administered, a Lycra swim cap was measured to find the DLPFC, which was marked on the cap. Earplugs were worn while receiving TMS [87].

To ensure that proper levels of TMS were applied, each participant’s MT was established before the experiment. The investigator applied supra-threshold TMS pulses to the contralateral abductor pollicis brevis muscle. This revealed the location of the strongest motor evoked potential (MEP). The coil of the TMS machine was held at a 45-degree angle from the hemispheric line. The coil was then moved around the head of the participant until locating the area that had the maximal peak-to-peak amplitude MEP. The MT of the participant was determined when a MEP of >50 μV was elicited after 50% of the TMS pulses had been delivered. This was performed by using the methods recommended by the International Federation of Clinical Neurophysiology [82]. TMS was administered throughout the experiment at 90% of the MT, and all MT measurements were performed using Trigno/DelSys.

**Figure 1:**
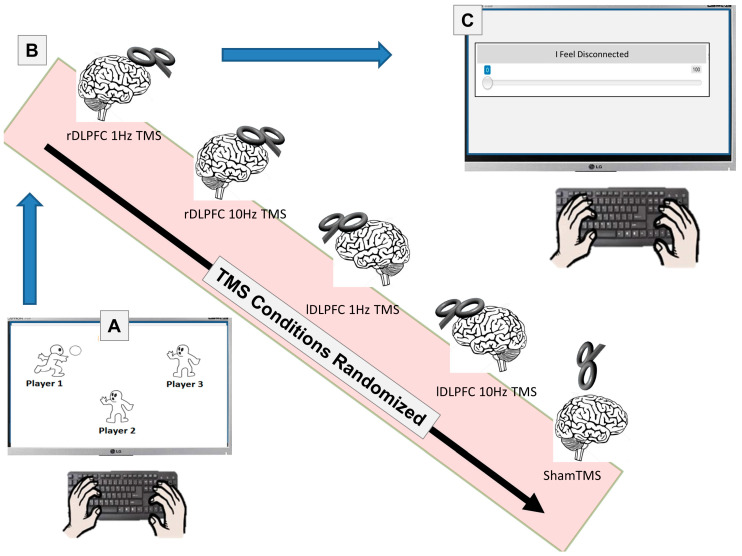
Overall procedure. Participants first played a brief game of Cyberball where they were socially excluded (**A**). Following the game (**B**), participants received TMS 5 times, in random order. Following each TMS session, the participants completed the reflective and reflexive Surveys (**C**).

Utilizing a Lenovo ThinkPad T490, the Cyberball program was administered to the participants once before any TMS. The number of players in the game was 3, with 2 of them being the computer and one of them being the participant. The number of throws was set to 20. The human participant received the ball 3 times at the beginning of the game, immediately followed by the exclusion scenario in which the player did not receive the ball for the rest of the game. There were no practice trials, and participants were instructed to click on the player they wanted to throw the ball to. Cyberball was played once per participant (Figure 1).

Following the establishment of the participant’s MT, TMS was administered to the DLPFC on both the left and right sides [84,88]. There was a total of 5 trials that were randomized: Sham for control, 10 Hz Left, 10 Hz Right, 1 Hz Left, and 1 Hz Right. For Sham, the TMS coil was held over the vertex (standard 10/20 system coordinates) at a 90-degree angle. TMS was discharged, but no pulses were delivered during the sham trial. An amount of 10 Hz TMS was administered for 6 s in 5 trains, which totaled 300 pulses, followed by a 20 s break between each of the trains [89,90,91]. The 1 Hz TMS was administered for 5 min in a single train, for a total of 300 pulses. Following the completion of each TMS session, the Testable stimuli were given to the participant (see above). A digitized analog scale was used with no markers to record responses. The scale was graded 0–100, though the numbers were hidden. The scales were completed 5 times after each TMS session.

This was a single blind study, meaning the participants were not aware of the hypothesis, while the researchers were aware.

### 2.5. Statistical Analyses

We performed a one-way repeated measures ANOVA to determine if there was a significant difference (*p* < 0.05) in the reflexive slider response, reflective slider response, and reaction times. If significance was found, we then performed a least significant difference (LSD) test to determine which brain conditions were significantly different.

Data on the 13 participants (N = 13) were analyzed using SPSS. We examined differences between reaction times (RT) and brain areas, along with reflexive and reflective answers to the questionnaire. Reflexive questions pertained to how the participants were feeling during the Cyberball game itself, whereas reflective questions were tailored to how the participants felt after the game.

## 3. Results

Was there a Reaction Time/Response Trade-off?

**Figure 2:**
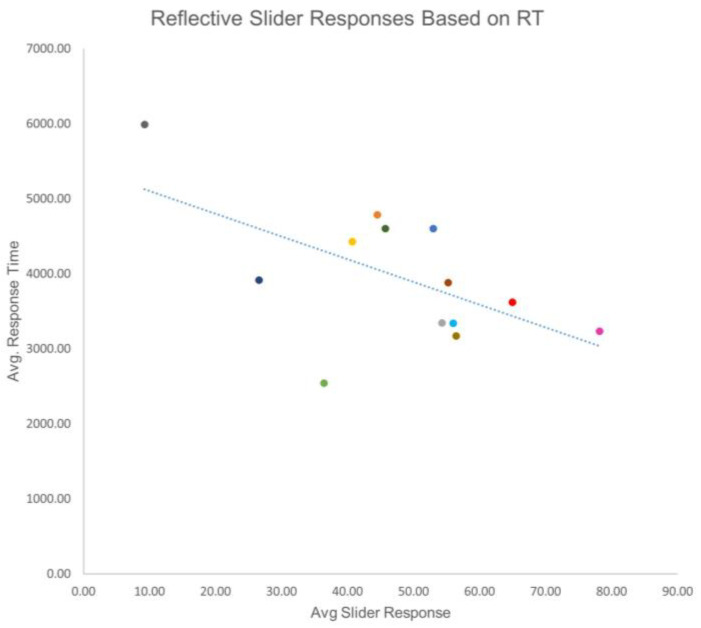
Reflective slider responses based on reaction time. This figure shows the relationship between the various reaction times and slider responses. There is a trend that shows quicker reaction times may attribute to higher scores regarding exclusion. Each color in Figure 2 represents a participant and corresponds with the same participant and color in Figure 3.

**Figure 3:**
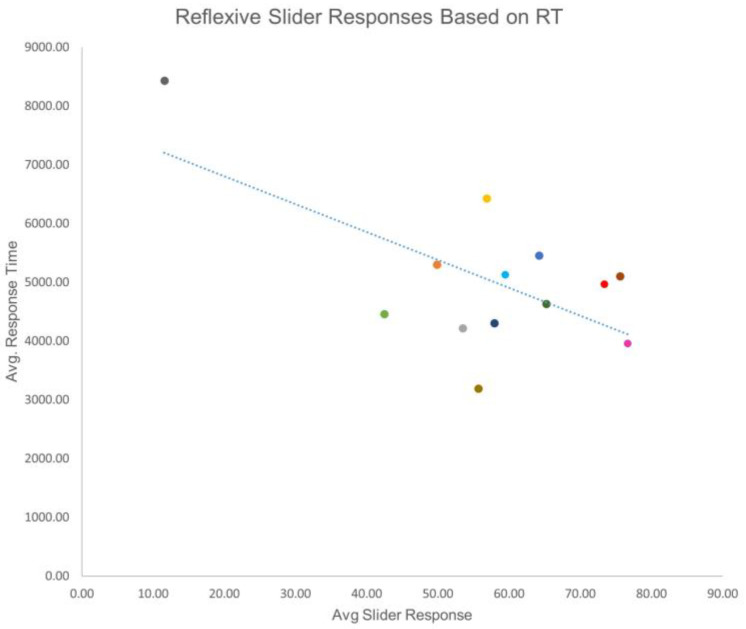
Reflexive slider responses based on reaction time. This figure shows the relationship between the various reaction times and slider responses. There is a trend that shows quicker reaction times may attribute to higher scores regarding exclusion, which is similar to Figure 1. Each color in Figure 2 represents a participant and corresponds with the same participant and color in Figure 1.

The mean reflective response was 47.79 (SD = 17.36), and the mean reflexive response was 57.10 (SD = 17.00). The mean reflective RT was 3956.80 (SD = 907.13), and the mean reflexive RT was 5043.56 (SD = 1291.36). To determine if there was a relationship between slider response and RT, a bivariate correlation was performed. In terms of reflective responses, it was found that as the slider response increased, RT decreased (r(12) = −0.579, *p* = 0.038). For reflexive responses, a similarly significant relationship was found (r(12) = −0.633, *p* = 0.020). These data indicated that there was a possibility that the less time a person contemplated a response, the more negative the response was likely to be (Figure 2 and Figure 3).

Were there Reflective Differences?

The five brain conditions were analyzed employing repeated measures ANOVAs for the various subscales. The reflective responses had no significant findings. For the reflective belonging responses, there were no significant differences (F(4,44) = 0.364, *p* = 0.833). Reflective certainty scales had no significance among treatments (F(4,44) = 0.465, *p* = 0.761). The control questions for the reflective scale had no significance (F(4,44) = 0.431, *p* = 0.786). Reflective mood scales also had no significance (F(4,44) = 0.635, *p* = 0.640). When it came to the perception of others, the reflective scale had no significance (F(4,44) = 0.349, *p* = 0.843). Anxiety, and more specifically social anxiety, had no significance when examining the reflective scale (F(4,44) = 0.238, *p* = 0.935). Lastly, for the reflective scale, self-esteem had no significance either (F(4,44) = 1.373, *p* = 0.259).

The insignificant results included mood (F(4,44) = 1.158, *p* = 0.342), manipulation check (F(4,44) = 0.134, *p* = 0.969), perception of others (F(4,44) = 1.310, *p* = 0.281), and anxiety (F(4,44) = 1.001, *p* = 0.417).

Were there Reflexive Differences?

**Figure 4:**
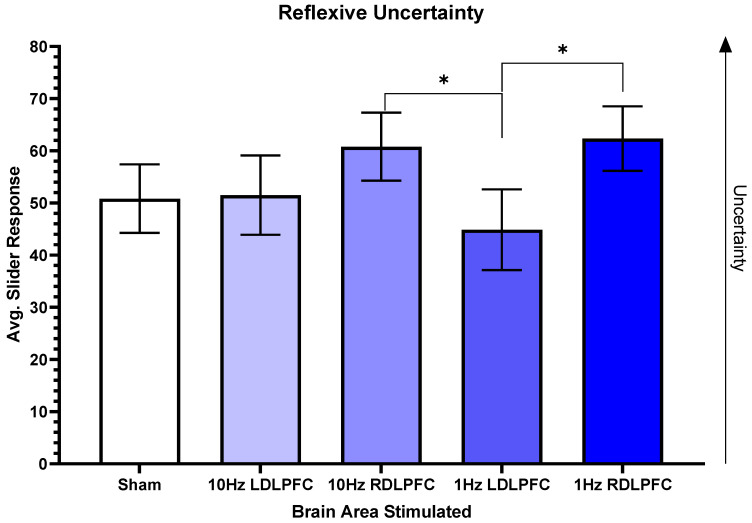
Reflexive uncertainty. This figure shows the average slider responses based on the region where TMS was performed. Higher scores correlated with more feelings of uncertainty. The significance between 10 Hz Right and 1 Hz Left is *p* = 0.046, whereas the significance between 1 Hz Left and 1 Hz Right is *p* = 0.017. Error bars display standard error, and all other comparisons are non-significant. * *p* < 0.05.

For the reflexive certainty scale, there was a trend for significance between two different areas. Overall, (F(4,44) = 2.430, *p* = 0.083) and the post-hoc tests revealed a significant difference between 10 Hz Right (M = 60.792 SD = 22.5343) compared to 1 Hz Left (M = 44.875 SD = 26.7719, t(12) = 2.22, *p* = 0.046, eta^2^ = 0.16). There was also a significant difference between 1 Hz Right (M = 62.333 SD = 21.4055) and 1 Hz Left (t(12) = 2.78, *p* = 0.017, eta^2^ = 0.19). Figure 4 shows the relationship between the different brain regions.

**Figure 5:**
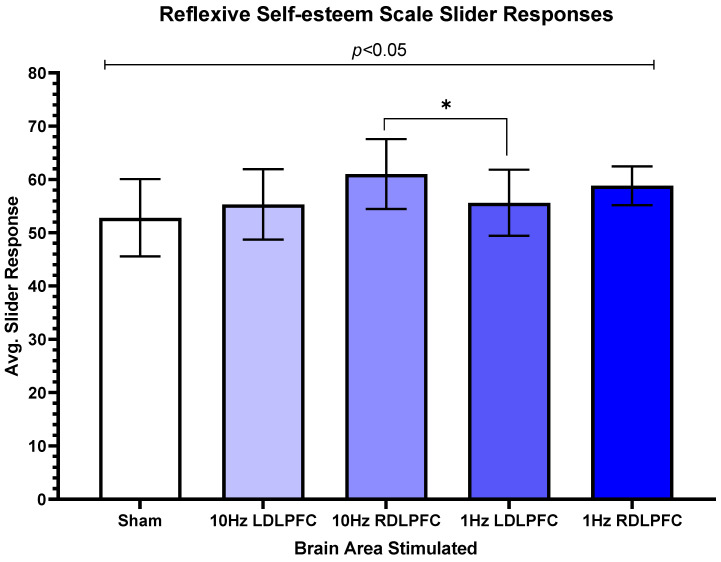
Reflexive self-esteem scale slider responses. The relationship between each area is shown based on a post-hoc test. The significant differences are for 10 Hz Right and 1 Hz Left. Standard error is plotted, and no other relationship is significant. Error bars display standard error, and all other comparisons are non-significant. * *p* < 0.05.

For reflexive self-esteem, there was also a significant difference (F(4,44) = 3.084, *p* = 0.025). The post-hoc tests revealed a trend between 10 Hz Left (M = 55.31, SD = 23.84) and 10 Hz Right (M = 61.04, SD = 23.68, t(12) = 1.95, *p* = 0.075). Figure 5 demonstrates differences between the brain regions stimulated by TMS.

**Figure 6:**
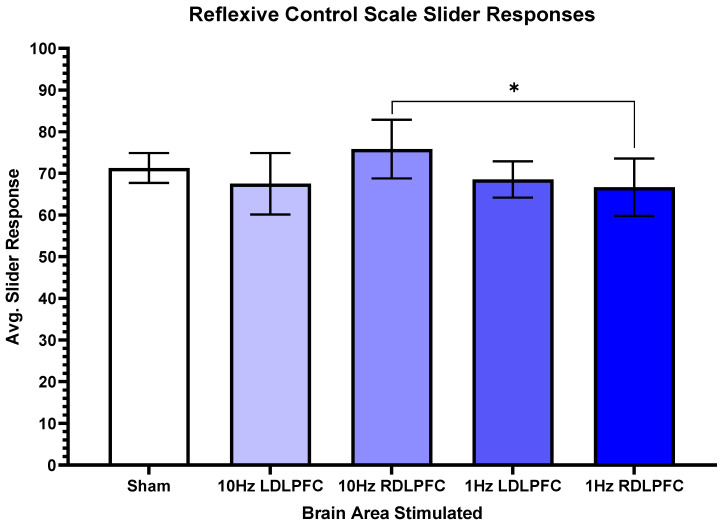
Reflexive control scale slider responses. This figure shows the relationship between the slider responses when asked about control over the game. There are significant differences in the 10 Hz Right and 1 Hz Right regions, *p* = 0.04. Standard error is plotted, and no other significant findings were reported. * *p* < 0.05.

For reflexive control, there was a significant difference (F(4,44) = 3.789, *p* = 0.011). Post-hoc tests revealed a significant difference between 10 Hz Right (M = 75.83, SD = 24.38) compared to 1 Hz Right (M = 66.67, SD = 23.84, t(12) = 2.30, *p* = 0.04). Figure 5 shows the effects of stimulating the right DLPFC with 10 Hz, and inhibition of the right DLPFC using 1 Hz (Figure 6).

## 4. Discussion

This study employed TMS to examine how social exclusion is perceived after manipulation of brain activity. The results demonstrated that the DLPFC, more specifically the right DLPFC, may be a key area when processing social exclusion. In comparison to the left hemisphere, manipulation of the right DLPFC appeared to have a greater effect on feelings of exclusion. Participants consistently reported feeling more excluded when the right DLPFC was excited, suggesting the possibility that there are increased feelings of social pain when the right DLPFC is manipulated.

Of interest is that quicker response times correlated with greater feelings of exclusion. There is a negative correlation between response times and slider responses. This suggests that a more painful feeling of exclusion could elicit a quicker response. Quicker response times could suggest a more honest initial answer, therefore resulting in more feelings of social exclusion. The higher values for both the reflexive and reflective questions indicate that there were greater feelings of exclusion. Higher scores for reflexive questions indicate stronger overall feelings of exclusion during the game, while higher reflective scores indicate greater overall feelings of exclusion after the game. Studies have shown that the right DLPFC is responsible for social decision making; therefore, manipulation of the right DLPFC in this study may correlate with those findings [40]. Conversely, given more time to think, participants had more positive responses. This relationship may be caused by demand characteristics. Demand characteristics occur when participants may be aware of what the researchers are investigating [92]. Participants may have known about the manufactured exclusion scenario and replied based on what they believed we were looking for. Participants in this situation may have responded more positively because they had more time to do so, enhancing their self-image and countering the idea of being excluded.

Our findings are in line with other exclusion experiments that suggest the prefrontal cortex is a key area when processing exclusion and other social experiences [20,93,94,95]. Makinodan and colleagues suggest that isolation events can instill both behavioral and cognitive changes in adults and may correlate with white matter alterations in the prefrontal cortex [96].

The reflexive certainty scale evaluated how participants felt about themselves during the Cyberball session. There were significant differences in slider responses when comparing both 10 Hz Right and 1 Hz Right TMS to 1 Hz Left TMS. When both exciting and inhibiting the right DLPFC, participants were more uncertain of themselves in the social event. This suggests that when exciting the right DLPFC, feelings of uncertainty about the social scenario increased. Conversely, it was seen that inhibition of the left DLPFC reduced feelings of exclusion compared to Sham, suggesting that inhibition of the left DLPFC reduced the effects of exclusion. Previous findings have indicated left PFC TMS reduces social exclusion [97,98]. Additionally, a previous study demonstrated that excitation of the right DLPFC can lead to a decrease in the ability to make decisions [99]. To elaborate, the previous study found a significant decrease in approach behavior when exciting the right DLPFC. Contrary to our current results, it was previously found that excitatory TMS of the left DLPFC improved certainty of social decision making [99]. Based on our data, it is suggested that inhibition of the left DLPFC may improve certainty in social decision making. Given that the left prefrontal cortex is typically associated with increased approach/positive emotions, inhibition would be anticipated to increase the emotional impacts of social exclusion. However, if decision making, or top-down regulation of emotion, via the right hemisphere mediates the impact of social exclusion, then inhibition of the left hemisphere might enable right hemisphere intrahemispheric coordination, resulting in the findings here. Future work could examine interhemispheric vs. intrahemispheric processing of the inter-relationship between cognitive appraisal and affect.

For the reflexive responses, self-esteem scores went beyond the concepts of certainty and instead emphasized the participants’ insecurities and sentiments. While overall feelings of exclusion were higher than average for each brain region, there was a significant difference between the 10 Hz Left and 10 Hz Right scores. Similar to the other subscales, the 10 Hz Right region had the strongest feelings of exclusion, suggesting that when increasing self-awareness through excitation, participants became more self-conscious. Specifically, the excitation of the right DLPFC was significantly higher than the excitation of the left DLPFC. Studies suggest that the DLPFC is recruited when judging oneself, and increased activity in the DLPFC leads to stronger criticism [100,101,102]. A further study found that highly self-critical people tend to have lower self-esteem [103]. These findings imply that the right DLPFC may be more responsible for negative self-view. Additionally, the DLPFC shows greater connectivity to other regions that are involved in negative feelings, such as the insula [104]. Moreover, the same connection is stronger in the right lateral prefrontal cortex, thus suggesting the right side is an area of significance [105,106]. It is possible, based on our current results, that the exclusion event may amplify feelings of self-criticism, in addition to negative affect more generally.

The last significant finding was the reflexive control scale. The results showed this subscale had the strongest feelings of exclusion. Since a majority of the responses were elevated compared to other groups, it indicates that manipulation of the DLPFC had an effect. Questions pertained to feeling in control and having influence over the game. Additionally, based on the questions asked, they could potentially relate to fairness. For example, there was a specific question that asked if participants felt the other players decided everything during the game. Based on our current findings, participants may have found the game less fair when the right DLPFC was excited. The current results could challenge previous findings that fairness is independent of the right DLPFC [44,45]. Additionally, the DLPFC is a key area of cognition control, so altering it will most likely have effects on responses [107,108]. Our current results suggest that inhibition of the left DLPFC reduces feelings of exclusion, as is seen in previous studies that relate to reduction in conditions such as depression [109].

Future studies should replicate these results using a larger sample size to examine replicability. Further studies could manipulate the length and parameters of the game. Here, the Cyberball game lasted for 20 throws, and future studies could increase the number of throws in order to potentially increase feelings of exclusion. Another limitation was the number of questions, which could be expanded if longer trains of TMS were delivered. Moreover, questions could be rephrased to account for demand characteristics. Additionally, the study only targeted the DLPFC, and exploring other brain regions may prove useful in elucidating the role of cortical areas in feelings of social exclusion. An area of interest could be the ventrolateral prefrontal cortex, as it was a common area associated with other exclusion and pain-related studies. Additionally, structures such as the insula or dACC should also be explored due to their involvement in the pain matrix.

## 5. Conclusions

In conclusion, our results demonstrate that manipulating activity in the DLPFC, especially the right DLPFC, significantly impacts social exclusion perceptions, suggesting this region plays a role in processing social rejection. Inhibition of the right DLPFC can amplify negative feelings and result in more social or emotional pain. The study also suggests that the left DLPFC can be involved in exclusion scenarios as well, especially in terms of the relationship with certainty during social situations. Unsurprisingly, these findings suggest that the processes examined here are lateralized, offering many avenues for future investigations. Note that overall results should be interpreted with caution due to the small sample size.

## Figures and Tables

**Table 1:** Reflexive questions asked during the experiment. These questions pertained to how participants felt during the game of Cyberball. Questions were pooled and displayed to the participants via Testable.

Questions	Category
I felt “disconnected” during the game.	Belonging
I felt “rejected” during the game.	Belonging
I felt like an outsider during the game.	Belonging
I felt good about myself during the game.	Self-esteem
My self-esteem was high during the game.	Self-esteem
I felt insecure during the game.	Self-esteem
I felt like I had control during the game.	Control
I felt the other players decided everything during the game.	Control
I felt powerful during the game.	Control
I felt uncertain about myself during the game.	Certainty
I did not know what I should be doing during the game.	Certainty
I felt unsure of what makes me who I am during the game.	Certainty
I felt friendly during the game.	Mood
I felt angry during the game.	Mood
I felt happy during the game.	Mood
I was ignored during the game.	Manipulation check
I was excluded during the game.	Manipulation check
I felt like I wanted to escape the game.	Anxiety
I felt like I wanted to leave the game.	Anxiety
I felt uneasy during the game.	Anxiety
I liked the other players.	Perception of others
I enjoyed playing with the others.	Perception of others
I was angry at the other players.	Perception of others

**Table 2:** Reflective questions asked during the experiment. These questions pertained to how participants felt after the game of Cyberball. Questions were pooled and displayed to the participants via Testable.

Questions	Category
I feel ‘disconnected’.	Belonging
I feel ‘rejected’.	Belonging
I feel like an outsider.	Belonging
I feel good about myself.	Self-esteem
My self-esteem is high.	Self-esteem
I feel insecure.	Self-esteem
I feel like I have control.	Control
I feel the other players decided everything.	Control
I feel powerful.	Control
I feel uncertain about myself.	Certainty
I do not know what I should be doing.	Certainty
I feel unsure of what makes me who I am.	Certainty
I feel friendly.	Mood
I feel angry.	Mood
I feel happy.	Mood
I would join another game.	Anxiety
I would sign up for another game.	Anxiety
I look forward to my next social event.	Anxiety
I like the other players.	Perception of others
I enjoy playing with the other players.	Perception of others
I am angry at the other players.	Perception of others

## Data Availability

Not applicable.
